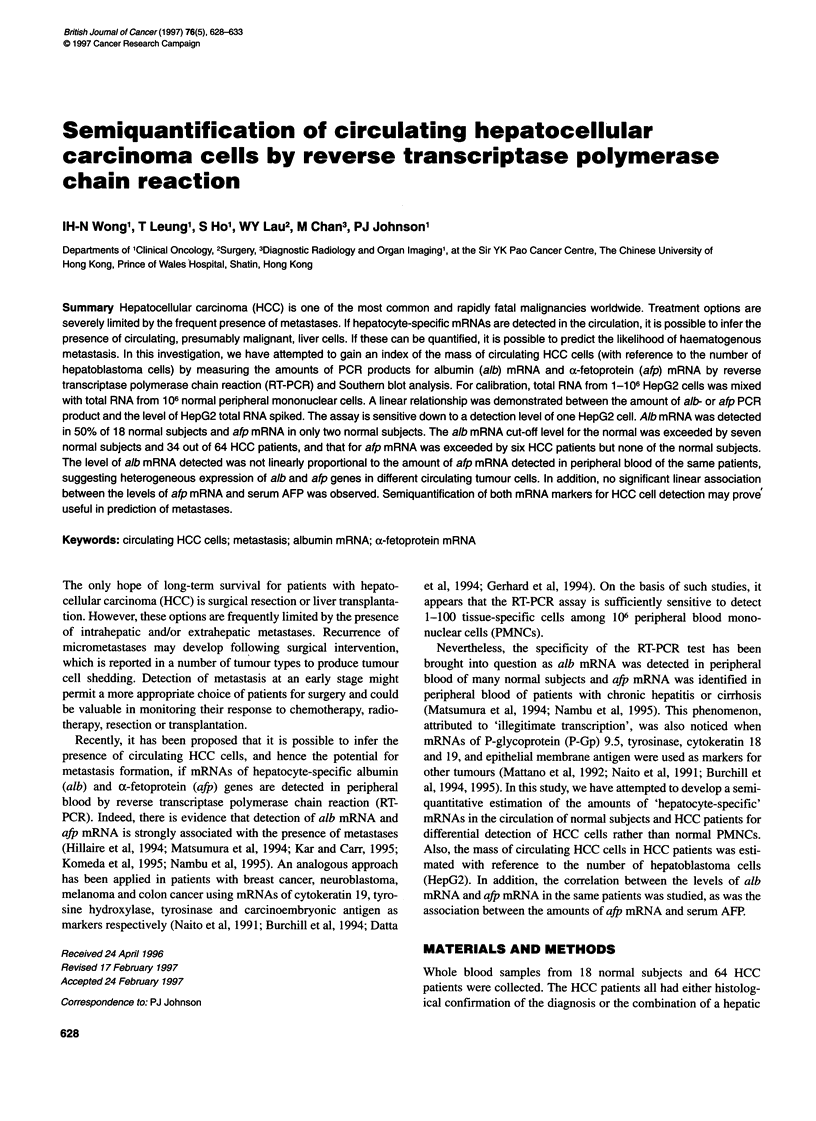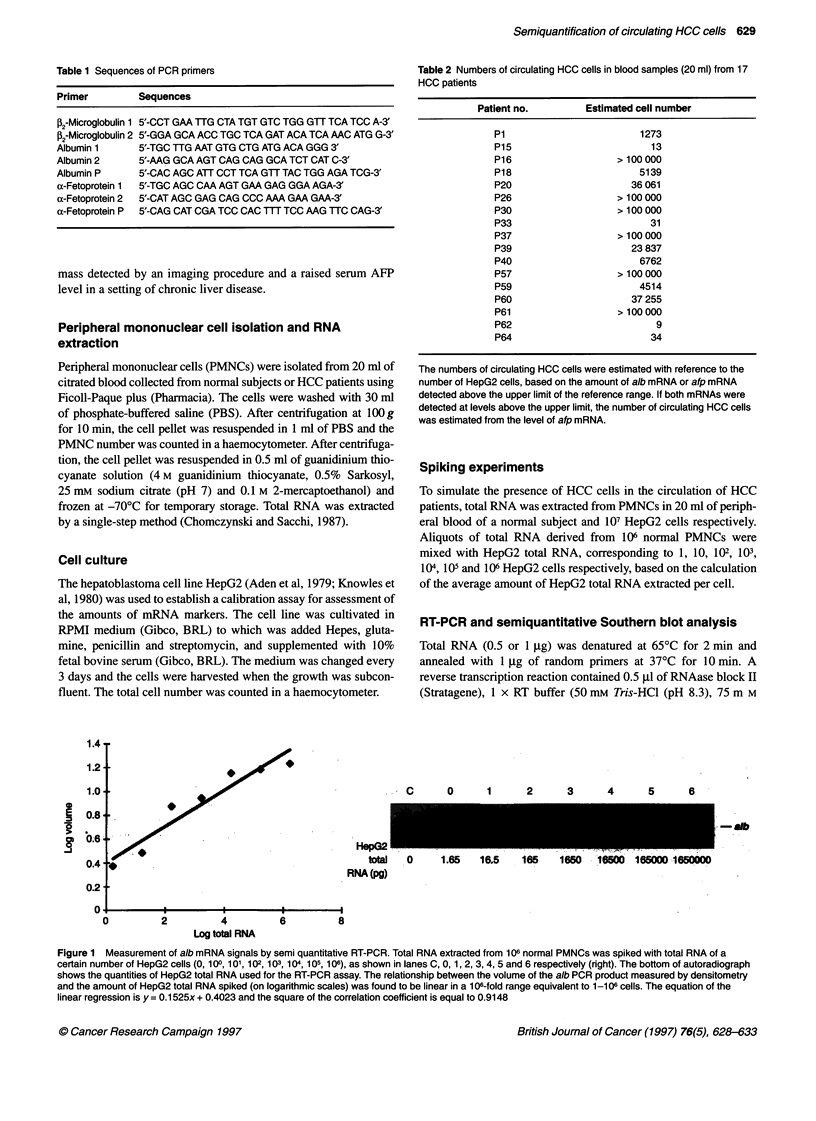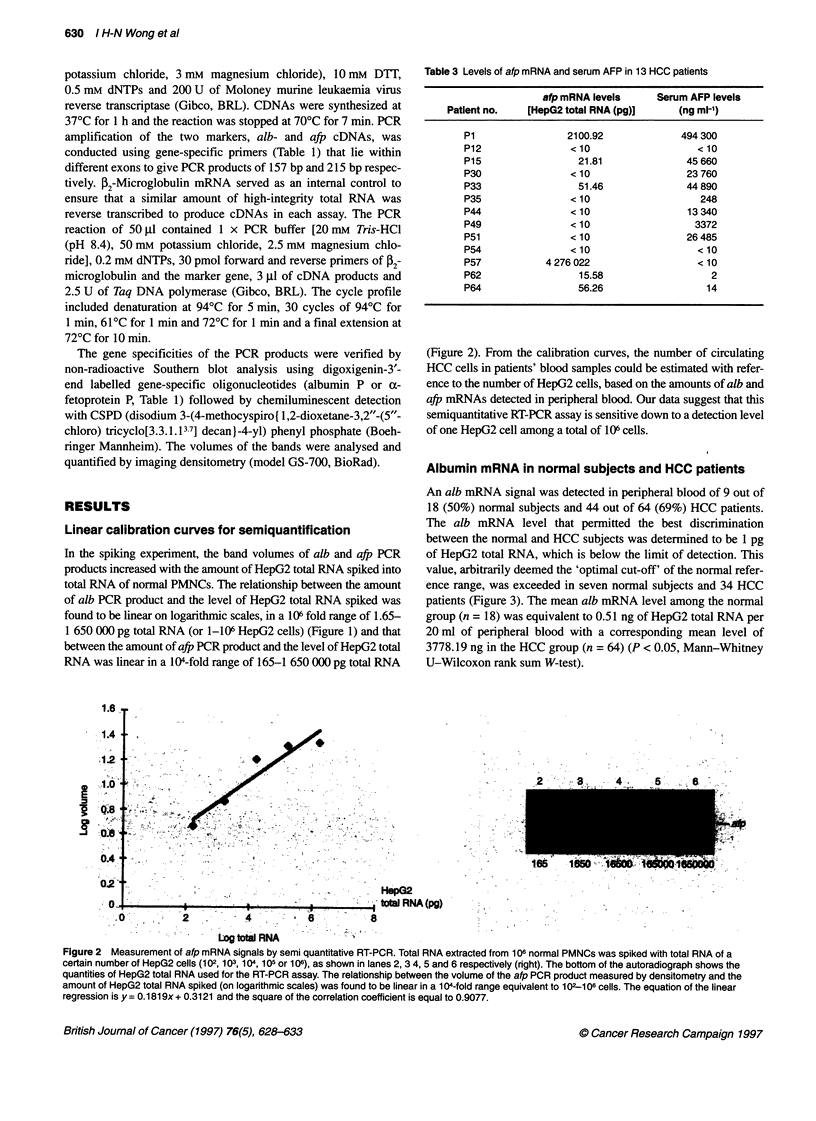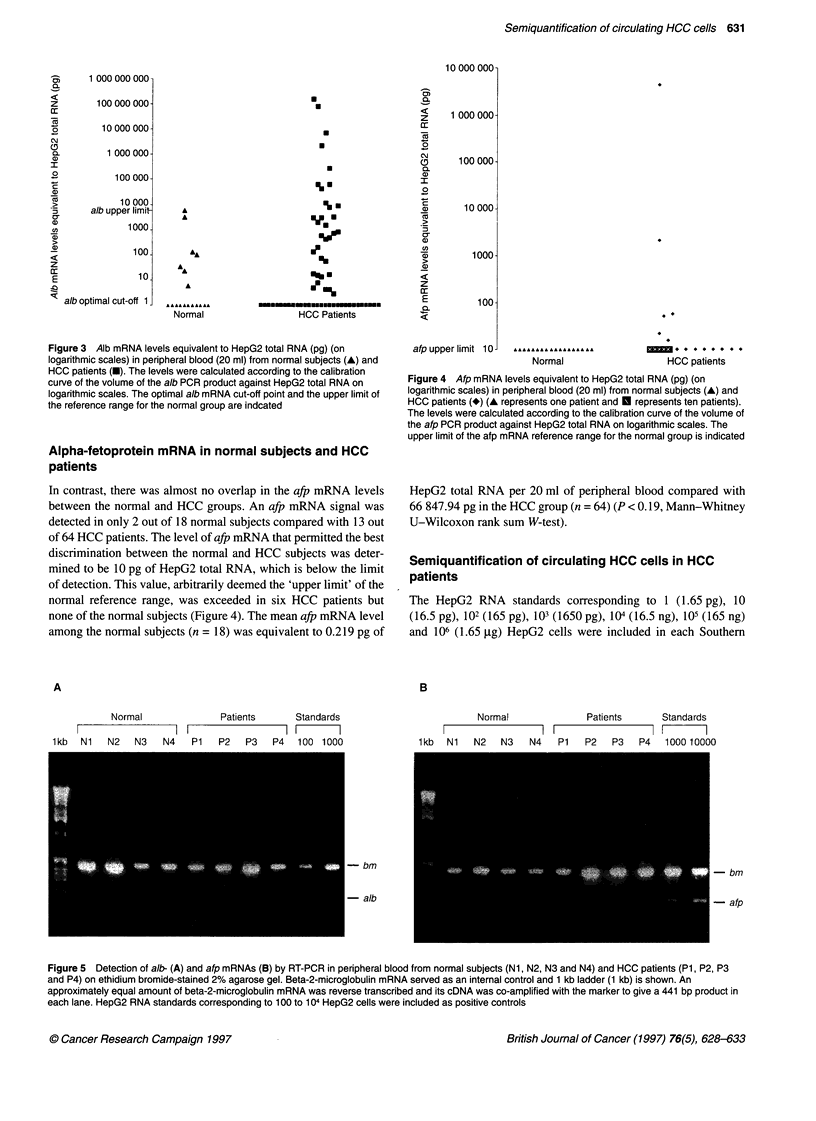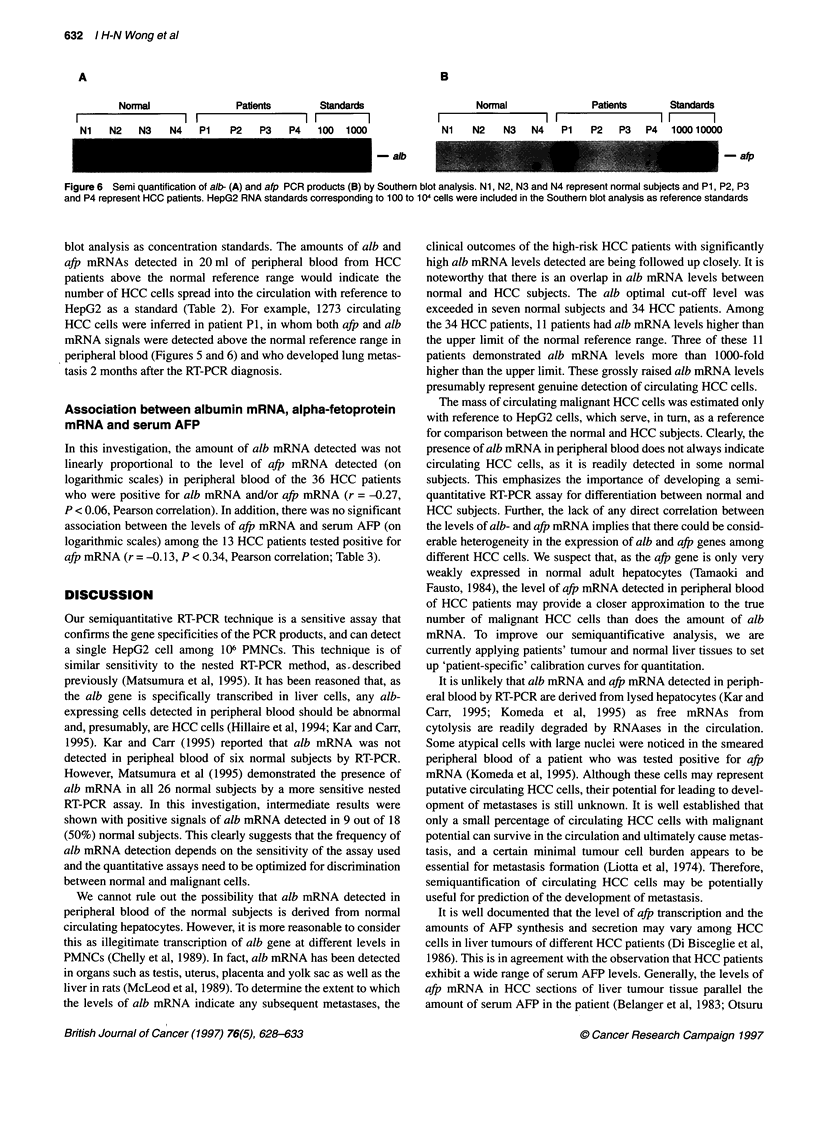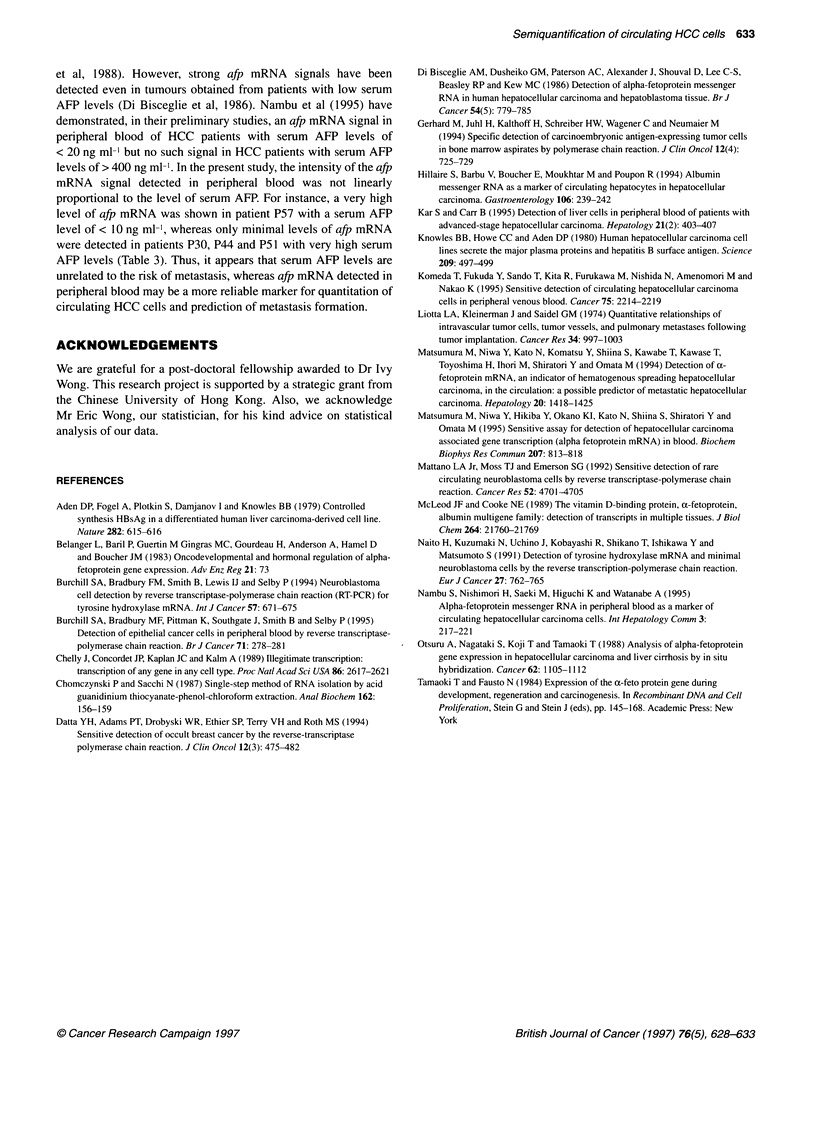# Semiquantification of circulating hepatocellular carcinoma cells by reverse transcriptase polymerase chain reaction.

**DOI:** 10.1038/bjc.1997.436

**Published:** 1997

**Authors:** I. H. Wong, T. Leung, S. Ho, W. Y. Lau, M. Chan, P. J. Johnson

**Affiliations:** Department of Clinical Oncology, Sir YK Pao Cancer Centre, The Chinese University of Hong Kong, Prince of Wales Hospital, Shatin.

## Abstract

**Images:**


British Journal of Cancer (1997) 76(5), 628–633
© 1997 Cancer Research Campaign

### Semiquantification of circulating hepatocellular carcinoma cells by reverse transcriptase polymerase chain reaction

**IH-N Wong[1], T Leung[1], S Ho[1], WY Lau[2], M Chan[3], PJ Johnson[1]**

Departments of [1]Clinical Oncology, [2]Surgery, [3]Diagnostic Radiology and Organ Imaging[1], at the Sir YK Pao Cancer Centre, The Chinese University of Hong Kong, Prince of Wales Hospital, Shatin, Hong Kong

**Summary** Hepatocellular carcinoma (HCC) is one of the most common and rapidly fatal malignancies worldwide. Treatment options are severely limited by the frequent presence of metastases. If hepatocyte-specific mRNAs are detected in the circulation, it is possible to infer the presence of circulating, presumably malignant, liver cells. If these can be quantified, it is possible to predict the likelihood of haematogenous metastasis. In this investigation, we have attempted to gain an index of the mass of circulating HCC cells (with reference to the number of hepatoblastoma cells) by measuring the amounts of PCR products for albumin (*alb*) mRNA and α-fetoprotein (*afp*) mRNA by reverse transcriptase polymerase chain reaction (RT-PCR) and Southern blot analysis. For calibration, total RNA from 1–$10^6$ HepG2 cells was mixed with total RNA from $10^6$ normal peripheral mononuclear cells. A linear relationship was demonstrated between the amount of *alb*- or *afp* PCR product and the level of HepG2 total RNA spiked. The assay is sensitive down to a detection level of one HepG2 cell. *Alb* mRNA was detected in 50% of 18 normal subjects and *afp* mRNA in only two normal subjects. The *alb* mRNA cut-off level for the normal was exceeded by seven normal subjects and 34 out of 64 HCC patients, and that for *afp* mRNA was exceeded by six HCC patients but none of the normal subjects. The level of *alb* mRNA detected was not linearly proportional to the amount of *afp* mRNA detected in peripheral blood of the same patients, suggesting heterogeneous expression of *alb* and *afp* genes in different circulating tumour cells. In addition, no significant linear association between the levels of *afp* mRNA and serum AFP was observed. Semiquantification of both mRNA markers for HCC cell detection may prove useful in prediction of metastases.

**Keywords:** circulating HCC cells; metastasis; albumin mRNA; α-fetoprotein mRNA

The only hope of long-term survival for patients with hepatocellular carcinoma (HCC) is surgical resection or liver transplantation. However, these options are frequently limited by the presence of intrahepatic and/or extrahepatic metastases. Recurrence of micrometastases may develop following surgical intervention, which is reported in a number of tumour types to produce tumour cell shedding. Detection of metastasis at an early stage might permit a more appropriate choice of patients for surgery and could be valuable in monitoring their response to chemotherapy, radiotherapy, resection or transplantation.

Recently, it has been proposed that it is possible to infer the presence of circulating HCC cells, and hence the potential for metastasis formation, if mRNAs of hepatocyte-specific albumin (*alb*) and α-fetoprotein (*afp*) genes are detected in peripheral blood by reverse transcriptase polymerase chain reaction (RT-PCR). Indeed, there is evidence that detection of *alb* mRNA and *afp* mRNA is strongly associated with the presence of metastases (Hillaire et al, 1994; Matsumura et al, 1994; Kar and Carr, 1995; Komeda et al, 1995; Nambu et al, 1995). An analogous approach has been applied in patients with breast cancer, neuroblastoma, melanoma and colon cancer using mRNAs of cytokeratin 19, tyrosine hydroxylase, tyrosinase and carcinoembryonic antigen as markers respectively (Naito et al, 1991; Burchill et al, 1994; Datta et al, 1994; Gerhard et al, 1994). On the basis of such studies, it appears that the RT-PCR assay is sufficiently sensitive to detect 1–100 tissue-specific cells among $10^6$ peripheral blood mononuclear cells (PMNCs).

Nevertheless, the specificity of the RT-PCR test has been brought into question as *alb* mRNA was detected in peripheral blood of many normal subjects and *afp* mRNA was identified in peripheral blood of patients with chronic hepatitis or cirrhosis (Matsumura et al, 1994; Nambu et al, 1995). This phenomenon, attributed to 'illegitimate transcription', was also noticed when mRNAs of P-glycoprotein (P-Gp) 9.5, tyrosinase, cytokeratin 18 and 19, and epithelial membrane antigen were used as markers for other tumours (Mattano et al, 1992; Naito et al, 1991; Burchill et al, 1994, 1995). In this study, we have attempted to develop a semiquantitative estimation of the amounts of 'hepatocyte-specific' mRNAs in the circulation of normal subjects and HCC patients for differential detection of HCC cells rather than normal PMNCs. Also, the mass of circulating HCC cells in HCC patients was estimated with reference to the number of hepatoblastoma cells (HepG2). In addition, the correlation between the levels of *alb* mRNA and *afp* mRNA in the same patients was studied, as was the association between the amounts of *afp* mRNA and serum AFP.

*Received 24 April 1996*
*Revised 17 February 1997*
*Accepted 24 February 1997*

*Correspondence to:* PJ Johnson

### MATERIALS AND METHODS

Whole blood samples from 18 normal subjects and 64 HCC patients were collected. The HCC patients all had either histological confirmation of the diagnosis or the combination of a hepatic

**Table 1** Sequences of PCR primers

| Primer | Sequences |
|---|---|
| $\beta_2$-Microglobulin 1 | 5′-CCT GAA TTG CTA TGT GTC TGG GTT TCA TCC A-3′ |
| $\beta_2$-Microglobulin 2 | 5′-GGA GCA ACC TGC TCA GAT ACA TCA AAC ATG G-3′ |
| Albumin 1 | 5′-TGC TTG AAT GTG CTG ATG ACA GGG 3′ |
| Albumin 2 | 5′-AAG GCA AGT CAG CAG GCA TCT CAT C-3′ |
| Albumin P | 5′-CAC AGC ATT CCT TCA GTT TAC TGG AGA TCG-3′ |
| α-Fetoprotein 1 | 5′-TGC AGC CAA AGT GAA GAG GGA AGA-3′ |
| α-Fetoprotein 2 | 5′-CAT AGC GAG CAG CCC AAA GAA GAA-3′ |
| α-Fetoprotein P | 5′-CAG CAT CGA TCC CAC TTT TCC AAG TTC CAG-3′ |

mass detected by an imaging procedure and a raised serum AFP level in a setting of chronic liver disease.

### Peripheral mononuclear cell isolation and RNA extraction

Peripheral mononuclear cells (PMNCs) were isolated from 20 ml of citrated blood collected from normal subjects or HCC patients using Ficoll-Paque plus (Pharmacia). The cells were washed with 30 ml of phosphate-buffered saline (PBS). After centrifugation at 100 *g* for 10 min, the cell pellet was resuspended in 1 ml of PBS and the PMNC number was counted in a haemocytometer. After centrifugation, the cell pellet was resuspended in 0.5 ml of guanidinium thiocyanate solution (4 M guanidinium thiocyanate, 0.5% Sarkosyl, 25 mM sodium citrate (pH 7) and 0.1 M 2-mercaptoethanol) and frozen at –70°C for temporary storage. Total RNA was extracted by a single-step method (Chomczynski and Sacchi, 1987).

### Cell culture

The hepatoblastoma cell line HepG2 (Aden et al, 1979; Knowles et al, 1980) was used to establish a calibration assay for assessment of the amounts of mRNA markers. The cell line was cultivated in RPMI medium (Gibco, BRL) to which was added Hepes, glutamine, penicillin and streptomycin, and supplemented with 10% fetal bovine serum (Gibco, BRL). The medium was changed every 3 days and the cells were harvested when the growth was subconfluent. The total cell number was counted in a haemocytometer.

**Table 2** Numbers of circulating HCC cells in blood samples (20 ml) from 17 HCC patients

| Patient no. | Estimated cell number |
|---|---|
| P1 | 1273 |
| P15 | 13 |
| P16 | > 100 000 |
| P18 | 5139 |
| P20 | 36 061 |
| P26 | > 100 000 |
| P30 | > 100 000 |
| P33 | 31 |
| P37 | > 100 000 |
| P39 | 23 837 |
| P40 | 6762 |
| P57 | > 100 000 |
| P59 | 4514 |
| P60 | 37 255 |
| P61 | > 100 000 |
| P62 | 9 |
| P64 | 34 |

The numbers of circulating HCC cells were estimated with reference to the number of HepG2 cells, based on the amount of *alb* mRNA or *afp* mRNA detected above the upper limit of the reference range. If both mRNAs were detected at levels above the upper limit, the number of circulating HCC cells was estimated from the level of *afp* mRNA.

### Spiking experiments

To simulate the presence of HCC cells in the circulation of HCC patients, total RNA was extracted from PMNCs in 20 ml of peripheral blood of a normal subject and $10^7$ HepG2 cells respectively. Aliquots of total RNA derived from $10^6$ normal PMNCs were mixed with HepG2 total RNA, corresponding to 1, 10, $10^2$, $10^3$, $10^4$, $10^5$ and $10^6$ HepG2 cells respectively, based on the calculation of the average amount of HepG2 total RNA extracted per cell.

### RT-PCR and semiquantitative Southern blot analysis

Total RNA (0.5 or 1 µg) was denatured at 65°C for 2 min and annealed with 1 µg of random primers at 37°C for 10 min. A reverse transcription reaction contained 0.5 µl of RNAase block II (Stratagene), 1 × RT buffer (50 mM *Tris*-HCl (pH 8.3), 75 m M

**Figure 1** Measurement of *alb* mRNA signals by semi quantitative RT-PCR. Total RNA extracted from $10^6$ normal PMNCs was spiked with total RNA of a certain number of HepG2 cells (0, $10^0$, $10^1$, $10^2$, $10^3$, $10^4$, $10^5$, $10^6$), as shown in lanes C, 0, 1, 2, 3, 4, 5 and 6 respectively (right). The bottom of autoradiograph shows the quantities of HepG2 total RNA used for the RT-PCR assay. The relationship between the volume of the *alb* PCR product measured by densitometry and the amount of HepG2 total RNA spiked (on logarithmic scales) was found to be linear in a $10^6$-fold range equivalent to 1–$10^6$ cells. The equation of the linear regression is $y = 0.1525x + 0.4023$ and the square of the correlation coefficient is equal to 0.9148

potassium chloride, 3 mM magnesium chloride), 10 mM DTT, 0.5 mM dNTPs and 200 U of Moloney murine leukaemia virus reverse transcriptase (Gibco, BRL). CDNAs were synthesized at 37°C for 1 h and the reaction was stopped at 70°C for 7 min. PCR amplification of the two markers, *alb*- and *afp* cDNAs, was conducted using gene-specific primers (Table 1) that lie within different exons to give PCR products of 157 bp and 215 bp respectively. $\beta_2$-Microglobulin mRNA served as an internal control to ensure that a similar amount of high-integrity total RNA was reverse transcribed to produce cDNAs in each assay. The PCR reaction of 50 μl contained 1 × PCR buffer [20 mM *Tris*-HCl (pH 8.4), 50 mM potassium chloride, 2.5 mM magnesium chloride], 0.2 mM dNTPs, 30 pmol forward and reverse primers of $\beta_2$-microglobulin and the marker gene, 3 μl of cDNA products and 2.5 U of *Taq* DNA polymerase (Gibco, BRL). The cycle profile included denaturation at 94°C for 5 min, 30 cycles of 94°C for 1 min, 61°C for 1 min and 72°C for 1 min and a final extension at 72°C for 10 min.

The gene specificities of the PCR products were verified by non-radioactive Southern blot analysis using digoxigenin-3′-end labelled gene-specific oligonucleotides (albumin P or α-fetoprotein P, Table 1) followed by chemiluminescent detection with CSPD (disodium 3-(4-methocyspiro{1,2-dioxetane-3,2″-(5″-chloro) tricyclo[3.3.1.1$^{3,7}$] decan}-4-yl) phenyl phosphate (Boehringer Mannheim). The volumes of the bands were analysed and quantified by imaging densitometry (model GS-700, BioRad).

### RESULTS

### Linear calibration curves for semiquantification

In the spiking experiment, the band volumes of *alb* and *afp* PCR products increased with the amount of HepG2 total RNA spiked into total RNA of normal PMNCs. The relationship between the amount of *alb* PCR product and the level of HepG2 total RNA spiked was found to be linear on logarithmic scales, in a $10^6$ fold range of 1.65–1 650 000 pg total RNA (or 1–$10^6$ HepG2 cells) (Figure 1) and that between the amount of *afp* PCR product and the level of HepG2 total RNA was linear in a $10^4$-fold range of 165–1 650 000 pg total RNA (Figure 2). From the calibration curves, the number of circulating HCC cells in patients' blood samples could be estimated with reference to the number of HepG2 cells, based on the amounts of *alb* and *afp* mRNAs detected in peripheral blood. Our data suggest that this semiquantitative RT-PCR assay is sensitive down to a detection level of one HepG2 cell among a total of $10^6$ cells.

**Table 3** Levels of *afp* mRNA and serum AFP in 13 HCC patients

| Patient no. | *afp* mRNA levels [HepG2 total RNA (pg)] | Serum AFP levels (ng ml$^{-1}$) |
|---|---|---|
| P1 | 2100.92 | 494 300 |
| P12 | < 10 | < 10 |
| P15 | 21.81 | 45 660 |
| P30 | < 10 | 23 760 |
| P33 | 51.46 | 44 890 |
| P35 | < 10 | 248 |
| P44 | < 10 | 13 340 |
| P49 | < 10 | 3372 |
| P51 | < 10 | 26 485 |
| P54 | < 10 | < 10 |
| P57 | 4 276 022 | < 10 |
| P62 | 15.58 | 2 |
| P64 | 56.26 | 14 |

### Albumin mRNA in normal subjects and HCC patients

An *alb* mRNA signal was detected in peripheral blood of 9 out of 18 (50%) normal subjects and 44 out of 64 (69%) HCC patients. The *alb* mRNA level that permitted the best discrimination between the normal and HCC subjects was determined to be 1 pg of HepG2 total RNA, which is below the limit of detection. This value, arbitrarily deemed the 'optimal cut-off' of the normal reference range, was exceeded in seven normal subjects and 34 HCC patients (Figure 3). The mean *alb* mRNA level among the normal group ($n$ = 18) was equivalent to 0.51 ng of HepG2 total RNA per 20 ml of peripheral blood with a corresponding mean level of 3778.19 ng in the HCC group ($n$ = 64) ($P < 0.05$, Mann–Whitney U–Wilcoxon rank sum $W$-test).

**Figure 2** Measurement of *afp* mRNA signals by semi quantitative RT-PCR. Total RNA extracted from $10^6$ normal PMNCs was spiked with total RNA of a certain number of HepG2 cells ($10^2$, $10^3$, $10^4$, $10^5$ or $10^6$), as shown in lanes 2, 3 4, 5 and 6 respectively (right). The bottom of the autoradiograph shows the quantities of HepG2 total RNA used for the RT-PCR assay. The relationship between the volume of the *afp* PCR product measured by densitometry and the amount of HepG2 total RNA spiked (on logarithmic scales) was found to be linear in a $10^4$-fold range equivalent to $10^2$–$10^6$ cells. The equation of the linear regression is $y = 0.1819x + 0.3121$ and the square of the correlation coefficient is equal to 0.9077.

 © Cancer Research Campaign 1997

**Figure 3** *A*lb mRNA levels equivalent to HepG2 total RNA (pg) (on logarithmic scales) in peripheral blood (20 ml) from normal subjects (▲) and HCC patients (■). The levels were calculated according to the calibration curve of the volume of the *alb* PCR product against HepG2 total RNA on logarithmic scales. The optimal *alb* mRNA cut-off point and the upper limit of the reference range for the normal group are indcated

**Figure 4** *Afp* mRNA levels equivalent to HepG2 total RNA (pg) (on logarithmic scales) in peripheral blood (20 ml) from normal subjects (▲) and HCC patients (◆) (▲ represents one patient and ▧ represents ten patients). The levels were calculated according to the calibration curve of the volume of the *afp* PCR product against HepG2 total RNA on logarithmic scales. The upper limit of the afp mRNA reference range for the normal group is indicated

### Alpha-fetoprotein mRNA in normal subjects and HCC patients

In contrast, there was almost no overlap in the *afp* mRNA levels between the normal and HCC groups. An *afp* mRNA signal was detected in only 2 out of 18 normal subjects compared with 13 out of 64 HCC patients. The level of *afp* mRNA that permitted the best discrimination between the normal and HCC subjects was determined to be 10 pg of HepG2 total RNA, which is below the limit of detection. This value, arbitrarily deemed the 'upper limit' of the normal reference range, was exceeded in six HCC patients but none of the normal subjects (Figure 4). The mean *afp* mRNA level among the normal subjects ($n$ = 18) was equivalent to 0.219 pg of HepG2 total RNA per 20 ml of peripheral blood compared with 66 847.94 pg in the HCC group ($n$ = 64) ($P < 0.19$, Mann–Whitney U–Wilcoxon rank sum *W*-test).

### Semiquantification of circulating HCC cells in HCC patients

The HepG2 RNA standards corresponding to 1 (1.65 pg), 10 (16.5 pg), $10^2$ (165 pg), $10^3$ (1650 pg), $10^4$ (16.5 ng), $10^5$ (165 ng) and $10^6$ (1.65 μg) HepG2 cells were included in each Southern

**Figure 5** Detection of *alb*- (**A**) and *afp* mRNAs (**B**) by RT-PCR in peripheral blood from normal subjects (N1, N2, N3 and N4) and HCC patients (P1, P2, P3 and P4) on ethidium bromide-stained 2% agarose gel. Beta-2-microglobulin mRNA served as an internal control and 1 kb ladder (1 kb) is shown. An approximately equal amount of beta-2-microglobulin mRNA was reverse transcribed and its cDNA was co-amplified with the marker to give a 441 bp product in each lane. HepG2 RNA standards corresponding to 100 to $10^4$ HepG2 cells were included as positive controls

© Cancer Research Campaign 1997 

**Figure 6** Semi quantification of *alb*- (**A**) and *afp* PCR products (**B**) by Southern blot analysis. N1, N2, N3 and N4 represent normal subjects and P1, P2, P3 and P4 represent HCC patients. HepG2 RNA standards corresponding to 100 to $10^4$ cells were included in the Southern blot analysis as reference standards blot analysis as concentration standards. The amounts of *alb* and *afp* mRNAs detected in 20 ml of peripheral blood from HCC patients above the normal reference range would indicate the number of HCC cells spread into the circulation with reference to HepG2 as a standard (Table 2). For example, 1273 circulating HCC cells were inferred in patient P1, in whom both *afp* and *alb* mRNA signals were detected above the normal reference range in peripheral blood (Figures 5 and 6) and who developed lung metastasis 2 months after the RT-PCR diagnosis.

### Association between albumin mRNA, alpha-fetoprotein mRNA and serum AFP

In this investigation, the amount of *alb* mRNA detected was not linearly proportional to the level of *afp* mRNA detected (on logarithmic scales) in peripheral blood of the 36 HCC patients who were positive for *alb* mRNA and/or *afp* mRNA ($r = -0.27$, $P < 0.06$, Pearson correlation). In addition, there was no significant association between the levels of *afp* mRNA and serum AFP (on logarithmic scales) among the 13 HCC patients tested positive for *afp* mRNA ($r = -0.13$, $P < 0.34$, Pearson correlation; Table 3).

### DISCUSSION

Our semiquantitative RT-PCR technique is a sensitive assay that confirms the gene specificities of the PCR products, and can detect a single HepG2 cell among $10^6$ PMNCs. This technique is of similar sensitivity to the nested RT-PCR method, as described previously (Matsumura et al, 1995). It has been reasoned that, as the *alb* gene is specifically transcribed in liver cells, any *alb*-expressing cells detected in peripheral blood should be abnormal and, presumably, are HCC cells (Hillaire et al, 1994; Kar and Carr, 1995). Kar and Carr (1995) reported that *alb* mRNA was not detected in peripheal blood of six normal subjects by RT-PCR. However, Matsumura et al (1995) demonstrated the presence of *alb* mRNA in all 26 normal subjects by a more sensitive nested RT-PCR assay. In this investigation, intermediate results were shown with positive signals of *alb* mRNA detected in 9 out of 18 (50%) normal subjects. This clearly suggests that the frequency of *alb* mRNA detection depends on the sensitivity of the assay used and the quantitative assays need to be optimized for discrimination between normal and malignant cells.

We cannot rule out the possibility that *alb* mRNA detected in peripheral blood of the normal subjects is derived from normal circulating hepatocytes. However, it is more reasonable to consider this as illegitimate transcription of *alb* gene at different levels in PMNCs (Chelly et al, 1989). In fact, *alb* mRNA has been detected in organs such as testis, uterus, placenta and yolk sac as well as the liver in rats (McLeod et al, 1989). To determine the extent to which the levels of *alb* mRNA indicate any subsequent metastases, the clinical outcomes of the high-risk HCC patients with significantly high *alb* mRNA levels detected are being followed up closely. It is noteworthy that there is an overlap in *alb* mRNA levels between normal and HCC subjects. The *alb* optimal cut-off level was exceeded in seven normal subjects and 34 HCC patients. Among the 34 HCC patients, 11 patients had *alb* mRNA levels higher than the upper limit of the normal reference range. Three of these 11 patients demonstrated *alb* mRNA levels more than 1000-fold higher than the upper limit. These grossly raised *alb* mRNA levels presumably represent genuine detection of circulating HCC cells.

The mass of circulating malignant HCC cells was estimated only with reference to HepG2 cells, which serve, in turn, as a reference for comparison between the normal and HCC subjects. Clearly, the presence of *alb* mRNA in peripheral blood does not always indicate circulating HCC cells, as it is readily detected in some normal subjects. This emphasizes the importance of developing a semi-quantitative RT-PCR assay for differentiation between normal and HCC subjects. Further, the lack of any direct correlation between the levels of *alb*- and *afp* mRNA implies that there could be considerable heterogeneity in the expression of *alb* and *afp* genes among different HCC cells. We suspect that, as the *afp* gene is only very weakly expressed in normal adult hepatocytes (Tamaoki and Fausto, 1984), the level of *afp* mRNA detected in peripheral blood of HCC patients may provide a closer approximation to the true number of malignant HCC cells than does the amount of *alb* mRNA. To improve our semiquantificative analysis, we are currently applying patients' tumour and normal liver tissues to set up 'patient-specific' calibration curves for quantitation.

It is unlikely that *alb* mRNA and *afp* mRNA detected in peripheral blood by RT-PCR are derived from lysed hepatocytes (Kar and Carr, 1995; Komeda et al, 1995) as free mRNAs from cytolysis are readily degraded by RNAases in the circulation. Some atypical cells with large nuclei were noticed in the smeared peripheral blood of a patient who was tested positive for *afp* mRNA (Komeda et al, 1995). Although these cells may represent putative circulating HCC cells, their potential for leading to development of metastases is still unknown. It is well established that only a small percentage of circulating HCC cells with malignant potential can survive in the circulation and ultimately cause metastasis, and a certain minimal tumour cell burden appears to be essential for metastasis formation (Liotta et al, 1974). Therefore, semiquantification of circulating HCC cells may be potentially useful for prediction of the development of metastasis.

It is well documented that the level of *afp* transcription and the amounts of AFP synthesis and secretion may vary among HCC cells in liver tumours of different HCC patients (Di Bisceglie et al, 1986). This is in agreement with the observation that HCC patients exhibit a wide range of serum AFP levels. Generally, the levels of *afp* mRNA in HCC sections of liver tumour tissue parallel the amount of serum AFP in the patient (Belanger et al, 1983; Otsuru

© Cancer Research Campaign 1997

et al, 1988). However, strong *afp* mRNA signals have been detected even in tumours obtained from patients with low serum AFP levels (Di Bisceglie et al, 1986). Nambu et al (1995) have demonstrated, in their preliminary studies, an *afp* mRNA signal in peripheral blood of HCC patients with serum AFP levels of < 20 ng $ml^{-1}$ but no such signal in HCC patients with serum AFP levels of > 400 ng $ml^{-1}$. In the present study, the intensity of the *afp* mRNA signal detected in peripheral blood was not linearly proportional to the level of serum AFP. For instance, a very high level of *afp* mRNA was shown in patient P57 with a serum AFP level of < 10 ng $ml^{-1}$, whereas only minimal levels of *afp* mRNA were detected in patients P30, P44 and P51 with very high serum AFP levels (Table 3). Thus, it appears that serum AFP levels are unrelated to the risk of metastasis, whereas *afp* mRNA detected in peripheral blood may be a more reliable marker for quantitation of circulating HCC cells and prediction of metastasis formation.

### ACKNOWLEDGEMENTS

We are grateful for a post-doctoral fellowship awarded to Dr Ivy Wong. This research project is supported by a strategic grant from the Chinese University of Hong Kong. Also, we acknowledge Mr Eric Wong, our statistician, for his kind advice on statistical analysis of our data.

### REFERENCES

Aden DP, Fogel A, Plotkin S, Damjanov I and Knowles BB (1979) Controlled synthesis HBsAg in a differentiated human liver carcinoma-derived cell line. *Nature* **282**: 615–616

Belanger L, Baril P, Guertin M Gingras MC, Gourdeau H, Anderson A, Hamel D and Boucher JM (1983) Oncodevelopmental and hormonal regulation of alpha-fetoprotein gene expression. *Adv Enz Reg* **21**: 73

Burchill SA, Bradbury FM, Smith B, Lewis IJ and Selby P (1994) Neuroblastoma cell detection by reverse transcriptase-polymerase chain reaction (RT-PCR) for tyrosine hydroxylase mRNA. *Int J Cancer* **57**: 671–675

Burchill SA, Bradbury MF, Pittman K, Southgate J, Smith B and Selby P (1995) Detection of epithelial cancer cells in peripheral blood by reverse transcriptase-polymerase chain reaction. *Br J Cancer* **71**: 278–281

Chelly J, Concordet JP, Kaplan JC and Kalm A (1989) Illegitimate transcription: transcription of any gene in any cell type. *Proc Natl Acad Sci USA* **86**: 2617–2621

Chomczynski P and Sacchi N (1987) Single-step method of RNA isolation by acid guanidinium thiocyanate-phenol-chloroform extraction. *Anal Biochem* **162**: 156–159

Datta YH, Adams PT, Drobyski WR, Ethier SP, Terry VH and Roth MS (1994) Sensitive detection of occult breast cancer by the reverse-transcriptase polymerase chain reaction. *J Clin Oncol* **12**(3): 475–482

Di Bisceglie AM, Dusheiko GM, Paterson AC, Alexander J, Shouval D, Lee C-S, Beasley RP and Kew MC (1986) Detection of alpha-fetoprotein messenger RNA in human hepatocellular carcinoma and hepatoblastoma tissue. *Br J Cancer* **54**(5): 779–785

Gerhard M, Juhl H, Kalthoff H, Schreiber HW, Wagener C and Neumaier M (1994) Specific detection of carcinoembryonic antigen-expressing tumor cells in bone marrow aspirates by polymerase chain reaction. *J Clin Oncol* **12**(4): 725–729

Hillaire S, Barbu V, Boucher E, Moukhtar M and Poupon R (1994) Albumin messenger RNA as a marker of circulating hepatocytes in hepatocellular carcinoma. *Gastroenterology* **106**: 239–242

Kar S and Carr B (1995) Detection of liver cells in peripheral blood of patients with advanced-stage hepatocellular carcinoma. *Hepatology* **21**(2): 403–407

Knowles BB, Howe CC and Aden DP (1980) Human hepatocellular carcinoma cell lines secrete the major plasma proteins and hepatitis B surface antigen. *Science* **209**: 497–499

Komeda T, Fukuda Y, Sando T, Kita R, Furukawa M, Nishida N, Amenomori M and Nakao K (1995) Sensitive detection of circulating hepatocellular carcinoma cells in peripheral venous blood. *Cancer* **75**: 2214–2219

Liotta LA, Kleinerman J and Saidel GM (1974) Quantitative relationships of intravascular tumor cells, tumor vessels, and pulmonary metastases following tumor implantation. *Cancer Res* **34**: 997–1003

Matsumura M, Niwa Y, Kato N, Komatsu Y, Shiina S, Kawabe T, Kawase T, Toyoshima H, Ihori M, Shiratori Y and Omata M (1994) Detection of α-fetoprotein mRNA, an indicator of hematogenous spreading hepatocellular carcinoma, in the circulation: a possible predictor of metastatic hepatocellular carcinoma. *Hepatology* **20**: 1418–1425

Matsumura M, Niwa Y, Hikiba Y, Okano KI, Kato N, Shiina S, Shiratori Y and Omata M (1995) Sensitive assay for detection of hepatocellular carcinoma associated gene transcription (alpha fetoprotein mRNA) in blood. *Biochem Biophys Res Commun* **207**: 813–818

Mattano LA Jr, Moss TJ and Emerson SG (1992) Sensitive detection of rare circulating neuroblastoma cells by reverse transcriptase-polymerase chain reaction. *Cancer Res* **52**: 4701–4705

McLeod JF and Cooke NE (1989) The vitamin D-binding protein, α-fetoprotein, albumin multigene family: detection of transcripts in multiple tissues. *J Biol Chem* **264**: 21760–21769

Naito H, Kuzumaki N, Uchino J, Kobayashi R, Shikano T, Ishikawa Y and Matsumoto S (1991) Detection of tyrosine hydroxylase mRNA and minimal neuroblastoma cells by the reverse transcription-polymerase chain reaction. *Eur J Cancer* **27**: 762–765

Nambu S, Nishimori H, Saeki M, Higuchi K and Watanabe A (1995) Alpha-fetoprotein messenger RNA in peripheral blood as a marker of circulating hepatocellular carcinoma cells. *Int Hepatology Comm* **3**: 217–221

Otsuru A, Nagataki S, Koji T and Tamaoki T (1988) Analysis of alpha-fetoprotein gene expression in hepatocellular carcinoma and liver cirrhosis by in situ hybridization. *Cancer* **62**: 1105–1112

Tamaoki T and Fausto N (1984) Expression of the α-feto protein gene during development, regeneration and carcinogenesis. In *Recombinant DNA and Cell Proliferation*, Stein G and Stein J (eds), pp. 145–168. Academic Press: New York

© Cancer Research Campaign 1997